# Supplementary material for: How does a partner’s motor variability affect joint action?
Source: PLoS One. 2020 Oct 29;15(10):e0241417. doi: 10.1371/journal.pone.0241417 (PMC7595416; doi:10.1371/journal.pone.0241417)
Supplement: S1 File — (PDF) [file pone.0241417.s001.pdf]

Interactions between factors obtained from analysis on participants' movement variability

The following texts include the interaction effects that were not significant in the 2x2x10 ANOVA performed on participants normalized movement variability on the horizontal and vertical dimension. All the significant interactions have been reported in the main manuscript.

Experiment 1

The interactions between axis-dimension and group did not reach a significance ($F(1,34)=0.144$, $p= 0.706$, $\eta^2 =0.004$) and neither did the interaction between repetition and axis-dimension ($F(9, 306)=0.750$, $p=0.663$, $\eta^2 =0.022$). The three-way interaction between the factors also failed to achieve significance ($F (9,306) = 1.120$, $p=0.0348$, $\eta^2 =0.032$).

Experiment 2

The interaction between repetition and group ($F(4,152) = 0.317$, $p= 0.866$, $\eta^2 = 0.008$) was not significant. The three-way interaction between the factors also failed to achieve significance ($F (4,152) = 0.308$, $p= 0.872$, $\eta^2 = 0.008$).

Experiment 3

The interaction between repetition and group ($F(4,148)= 0.189$, $p= 0.944$, $\eta^2 =0.005$) was not significant and neither was the interaction between axis-dimension and group ($F(1,37)= 0.876$, $p= 0.355$, $\eta^2 = 0.023$). The interaction between axis-dimension and repetition ($F(4,148)= 1.761$, $p= 0.140$, $\eta^2 =0.045$) was also not significant, as well as the three-way interaction between the factors also failed to achieve significance ($F (4,148) = 0.437$, $p=0.225$, $\eta^2 =0.037$).
