# Supplementary material for: How does a partner’s motor variability affect joint action?
Source: PLoS One. 2020 Oct 29;15(10):e0241417. doi: 10.1371/journal.pone.0241417 (PMC7595416; doi:10.1371/journal.pone.0241417)
Supplement: S2 File — (PDF) [file pone.0241417.s002.pdf]

Analysis of confederate's spatial accuracy performance

We analysed the confederate's data to understand how the confederate modulated their movements over time, in all the three experiments.

Experiment 1

The *spatial error (cm)* of the confederate's performance was subjected to a mixed 2x10 ANOVA with Group (High Variability (HV) and Low Variability (LV) group) as the between-subject factor and Repetitions (R1-R10) as the within-subject factor. Greenhouse-Geisser corrected values were used for factors that violated Mauchly's sphericity test. The spatial error for all 10 repetitions of the sequences was calculated and averaged across all 8 blocks. The results are as follows:

The ANOVA on the confederate's data with a Greenhouse-Geisser correction (ε =605) revealed a main effect of the repetitions, indicating that confederates in both groups showed reduction of spatial error over time (F (9,306) = 12.169, p<0.0001, η^2 = 0.264). The analysis also showed a significant main effect of group, with the HV group (Mean= 1.377, SE = 0.092) having significantly larger errors than the LV group (Mean = 1.032, SE = 0.092), (F (1, 34) =7.075, p=0.012, η^2 = 0.172). The interaction between the two factors was also significant (F (9, 306) = 7.646, p< 0.0001, η^2 = 0.184), where R4,R5,R6 and R7 of HV group was significantly different from all other levels (all *p*s <0.05).

Experiment 2

The s*patial error (cm)* of the confederate's performance was subjected to a mixed 2x5ANOVA with Group (High Variability (HV) and Low Variability (LV) group) as the between-subject factor and Repetitions (R1-R5) as the within-subject factor. Greenhouse-Geisser corrected values were used for factors that violated Mauchly's sphericity test. The spatial error for all 5 repetitions of the sequences was calculated and averaged across all 8 blocks. The results are as follows:

The ANOVA with a Greenhouse-Geisser correction (ε =0.364) revealed a main effect of Repetition, indicating that confederates in both groups reduced their spatial error over time ($F (4, 152) = 41.260$, $p< 0.0001$, $\eta^2 = 0.521$). However, spatial error of the HV group (Mean= 0.625, SE= 0.028) did not differ from that of the LV (Mean= 0.550, SE= 0.028) group ($F (1, 38) = 3.578$, $p = 0.066$, $\eta^2 = 0.086$). The interaction between the factors was significant ($F (4,152) = 4.686$, $p= 0.022$, $\eta^2 = 0.110$). Post-hoc analysis revealed that the interaction was driven by R1 and R2 of HV group which was significantly different from that of the LV group.

Experiment 3

The confederate's data was processed and analysed as in Experiment 2. The results are as follows:

The ANOVA with a Greenhouse-Geisser correction (ε =0.673) revealed a main effect of Repetition, confirming that confederates in both groups learned to reduce their spatial error over time ($F (4, 148) = 6.010$, $p< 0.0001$, $\eta^2= 0.140$). There was no main effect of Group ($F (1, 37) = 2.90$, $p = 0.097$, $\eta^2 = 0.073$), indicating that spatial error did not differ significantly between HV group (Mean= 1.152, SE= 0.121) and LV group (Mean= 0.864, SE= 0.118). The interaction between the factors was significant ($F (4,148) = 7.235$, $p <0.001$, $\eta^2 = 0.164$). The post-hoc revealed that R3 of HV group was significantly different from the other levels ($p=0.005$).

Taken together, the results show an overall learning effect, as confederate also improves her performance between first and last repetitions across experiments. On one hand, this is a desired pattern as it adds to the ecological validity of the motor interaction between co-actors. On the other hand, this could be a confound with respect to the source of participants' learning. In fact, participants could be simply mimicking the confederate's movements without learning. But this cannot be the case in the current study, as participants and confederate never perform the same movements, neither do they perform similar trial by trial movements, nor do they cover the same distance (therefore experience the same pull). Hence, participants would not improve their performance if they were only mimicking the confederate's movements (see supporting information S5 File, pg. 7-11 for further analyses and discussion).
