# Supplementary material for: How does a partner’s motor variability affect joint action?
Source: PLoS One. 2020 Oct 29;15(10):e0241417. doi: 10.1371/journal.pone.0241417 (PMC7595416; doi:10.1371/journal.pone.0241417)
Supplement: S3 File — (PDF) [file pone.0241417.s003.pdf]

Repeated measures correlations (rmcorr) on LV group data

Experiment 1

A repeated measures correlation (rmcorr) was computed for R1 and R10 separately, to determine the relationship between the force perturbation experienced and the spatial error in the LV group. The correlation analysis did not reveal any significant correlations neither at R1 (r_{rm}(142)=-0.082, p =0.328, 95% CI= -0.2434504 0.083848) nor at R10 (r_{rm}(179)=0.019, p =0.794, 95% CI= -0.127582 0.1656539). A scatter plot (see S3 Fig 1) summarizes the results.

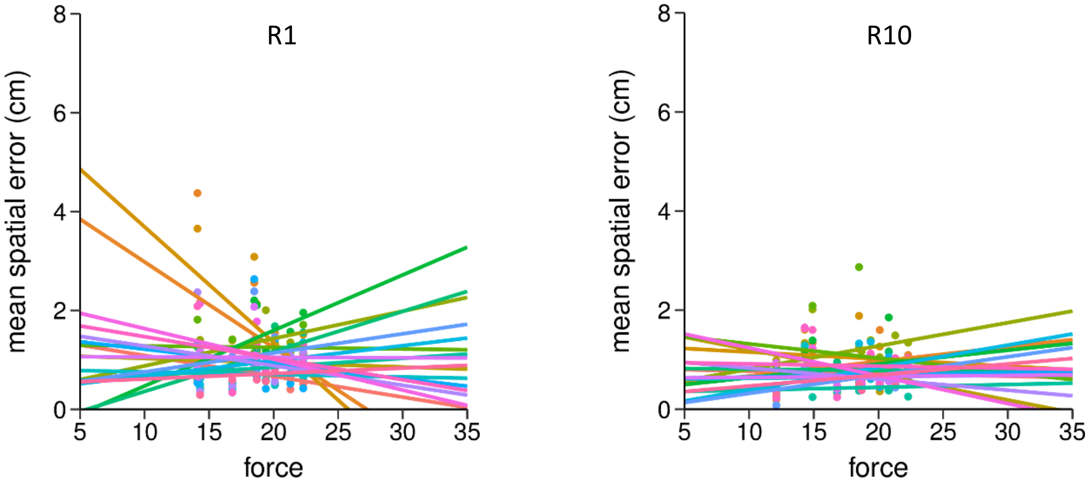

S3 Fig 1. Results of Experiment 1. Repeated measures correlational analysis between the force perturbation experienced and the spatial error of the participants in the LV group, for R1 and R10 separately. Each participant's data and corresponding rmcorr fit lines are shown in different colors.

Experiment 2

A repeated measures correlation (rmcorr) was computed for R1 and R5 separately, to determine the relationship between the force perturbation experienced and the spatial error in the LV group.

The correlation analysis did not reveal any significant correlations neither at R1 ($r_{rm}(59)=0.085$, p =0.510, 95% CI= -0.1740236, 0.3346091) nor at R10 ($r_{rm}(59)=0.039$, p =0.761, 95% CI= -0.2186034, 0.2928067). A scatter plot (see S3 Fig 2) summarizes the results.

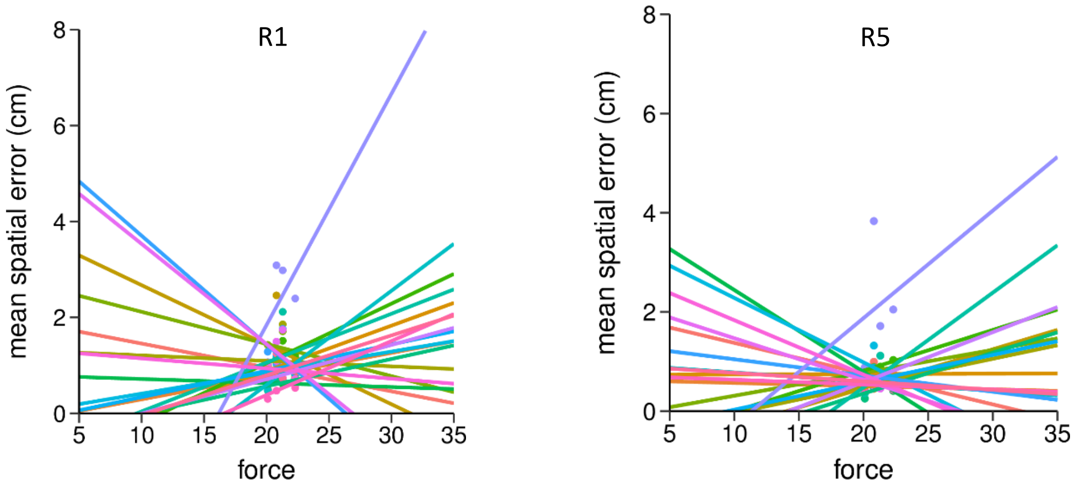

S3 Fig 2. Results of Experiment 2. Repeated measures correlational analysis between the force perturbation experienced and the spatial error of the participants in the LV group, for R1 and R 5 separately. Each participant's data and corresponding rmcorr fit lines are shown in different colors.

Experiment 3

A repeated measures correlation (rmcorr) was computed for R1 and R5 separately, to determine the relationship between the force perturbation experienced and the spatial error in the LV group. The correlation analysis revealed a significant positive correlation at R1 ($r_{rm}(99)= 0.217$, p =0.029; 95% CI= 0.02048916, 0.3974088) and no correlation at R5 (0.029, p = 0.770; 95% CI= -0.1690251, 0.2254713). A scatter plot (see S3 Fig 3) summarizes the results.

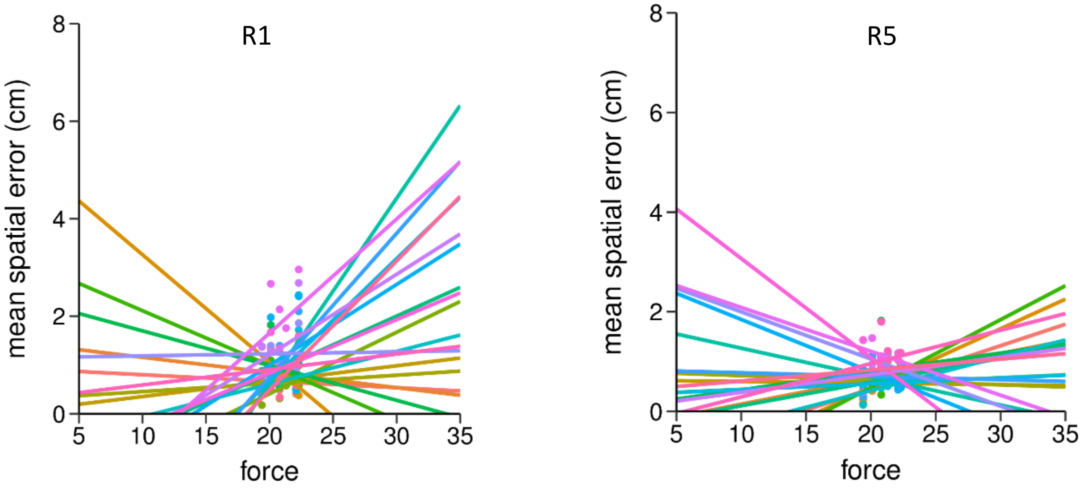

S3 Fig 3. Results of Experiment 3. Repeated measures correlational analysis between the force perturbation experienced and the spatial error of the participants in the LV group, for R1 and R5 separately. Each participant's data and corresponding rmcorr fit lines are shown in different colors.
