# Supplementary material for: How does a partner’s motor variability affect joint action?
Source: PLoS One. 2020 Oct 29;15(10):e0241417. doi: 10.1371/journal.pone.0241417 (PMC7595416; doi:10.1371/journal.pone.0241417)
Supplement: S4 File — (PDF) [file pone.0241417.s004.pdf]

# Participants' movement time analysis

Actors in our experiments were instructed to synchronize with an isochronous sequence of tones that dictated moving within the 1000 ms cue period of the targets (this has been clarified under the Experimental Timeline of each experiment in the manuscript). Thus, a speed-accuracy trade-off seems unlikely in the present task. Nevertheless, we performed additional analyses on the movement times of the participants. We here report the analysis of participant's movement times (defined as the time from target to target). Movement time was subjected to a repeated measures ANOVA with Group (2) as between-subject factor and Repetitions (10 or 5) as within-subject factor. The analysis reveals that in all the three experiments, participants show a main effect of repetition, with the movement time at R1 always higher than the final repetition. Taking these results, together with the reduction of spatial error observed in the main analysis of spatial error (submitted in the manuscript) it is evident that, while participants learn to get more accurate in their movements, they also get faster in all three experiments. If there was a speed-accuracy trade-off, the change in movement time and spatial error over repetitions should have followed opposite trends, which is not what we observe in our data. The analysis on the movement times are as follows:

## Experiment 1

The 2 x 10 ANOVA on the participant's movement time revealed a main effect of the repetitions, indicating that participants in both groups reduced their movement time over repetitions ($F(9,306) = 6.612$, $p<0.0001$, $\eta^2 = 0.163$, see S4 Fig 1). Post hoc revealed that the first repetition R1 was significantly different from all other repetitions (mean= 0.842, SE= 0.004, all *p*s< 0.005)The main effect of group (HV group: mean=0.832, SE=0.006 and LV group: mean= 0.829, SE= 0.006) failed

to reach a significance ($F(1,34)= 0.103$, $p= 0.7495$, $\eta^2 = 0.003$. Even though the interaction between the two factors was significant ($F (9, 306) = 2.601$, $p= 0.007$, $\eta^2 = 0.071$), post-hoc analysis did not reveal any significant differences (all *p*s >0.1).

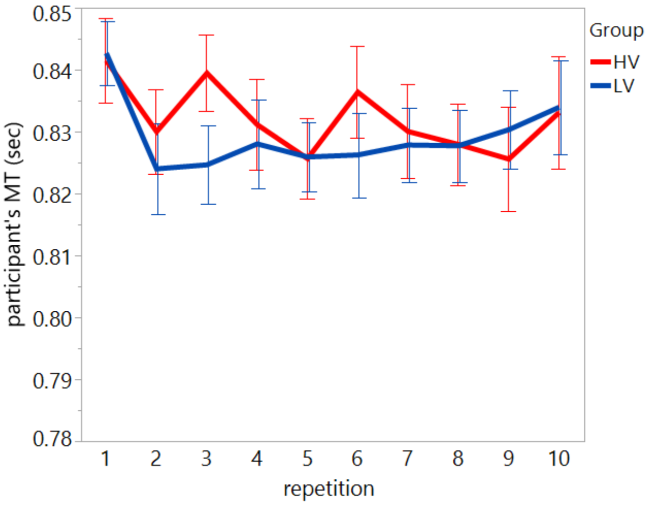


**S4 Fig 1.** Analysis of participants' movement time in Experiment 1.

# Experiment 2

The 2 x 5 ANOVA on the participant's movement time revealed a main effect of Repetition, indicating that participants in both groups showed reduction of movement time across repetitions ($F (4,152) = 31.447$, $p<0.0001$, $\eta^2 =0.453$, see S4 Fig 2). Post-hoc analyses revealed that R1 (mean= 0.827, SE=0.005) was significantly higher than all other repetitions (all *p*s<0.0001).The main effect of group (HV group: mean=0.812, SE=0.006 and LV group: mean= 0.806, SE= 0.006) was not significant ($F (1,38) = 0.437$, $p= 0.513$, $\eta^2 =0.011$). The interaction between the two factors was significant ($F (4,152) = 5.838$, $p< 0.001$, $\eta^2 = 0.133$). Post-hoc revealed that the HV group had a higher movement time at R1 (mean= 0.837, SE= 0.007) compared to the LV group (mean= 0.816, SE= 0.007).

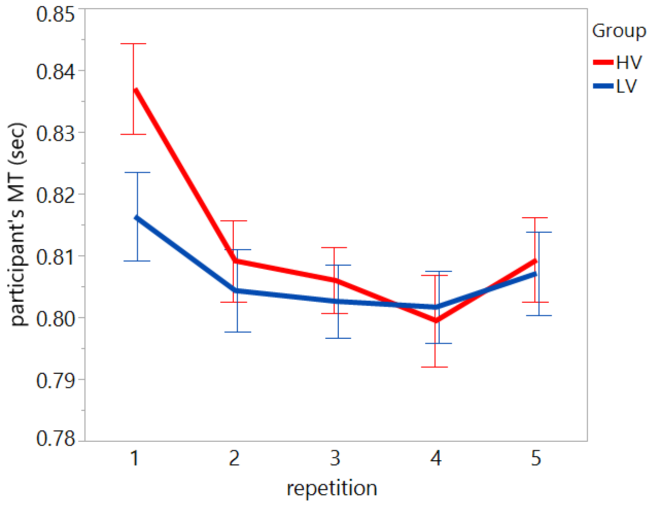


**S4 Fig 2.** Analysis of participants' movement time in Experiment 2.

# Experiment 3

The 2 x 5 ANOVA on the participant's movement time revealed a main effect of Repetition, indicating that participants in both groups showed reduction of movement time over repetition (F (4,148) =7.630, p<0.0001, $\eta^2$ =0.171, see S4 Fig 3). Post-hoc revealed that movement time at R1 (mean= 0.826, SE= 0.004) was significantly higher compared to R2-R4 (all *p*s <0.006). The main effect of group (HV group: mean= 0.818, SE=0.008 and LV group: mean= 0.820, SE= 0.008) did not reach a significance (F(1,37)= 0.099, p= 0.755, $\eta^2$ =0.003). The interaction between the two factors was significant (F (4,148) = 2.665, p= 0.034, $\eta^2$ = 0.067). However, post-hoc tests did not reach a significance (all *p*s>0.16).

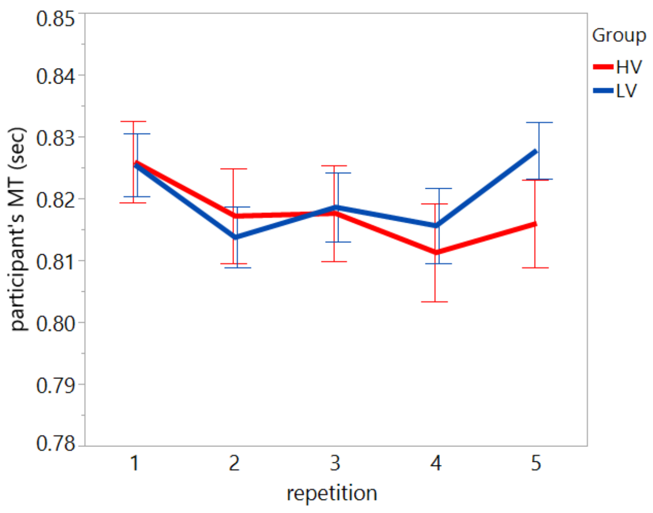


**S4 Fig 3.** Analysis of participants' movement time in Experiment 3.
