# Supplementary material for: How does a partner’s motor variability affect joint action?
Source: PLoS One. 2020 Oct 29;15(10):e0241417. doi: 10.1371/journal.pone.0241417 (PMC7595416; doi:10.1371/journal.pone.0241417)
Supplement: S5 File — (PDF) [file pone.0241417.s005.pdf]

Miscellaneous analysis

Analysis of Repetition 1 of Block 1 on consecutive targets

In the spatial error analyses submitted in the manuscript, the HV and LV group performances are already different at R1 in Experiment 1, while they are not different in Experiment 2 and 3. Since, predictability of confederate's movements can only be experienced after the first repetition (as predictability is the order in which the forces are delivered), one could assume that there is a potential confound driving the difference between groups at R1 in Experiment 1. To address this concern, we analysed the average spatial error performance of the first repetition (R1) of the **first block -** which includes 8 different target locations. This represents the first instance of participants' performance of a sequence (8 targets) in the presence of force perturbations. Since the first repetition of the first block is unpredictable regardless of the experimental condition, one would expect no difference in performance for all participants in both groups and across the three experiments. We subjected the participants' spatial error to a 2 x 8 mixed ANOVA with Group (2) as the between-subjects factor and Target Number (8) as the within-subjects factor.

Experiment 1

The ANOVA, with Greenhouse-Geisser correction ($\varepsilon = 0.435$), on the participant's spatial error at R1 of Block 1 revealed a main effect of the Targets, indicating that participants reduced their spatial error across consecutive targets ($F(7, 238) = 10.047$, $p<0.0001$, $\eta^2 = 0.228$, see S5 Fig 1). The main effect of Group (HV group: mean= 2.601, SE= 0.233 and LV group: mean= 2.114, SE=

0.233) failed to reach a significance ($F(1,34)= 2.183$, $p= 0.149$, $\eta^2 =0.060$). The interaction between the two factors was also not significant ($F (7,238) = 1.901$, $p= 0.070$, $\eta^2 =0.053$).

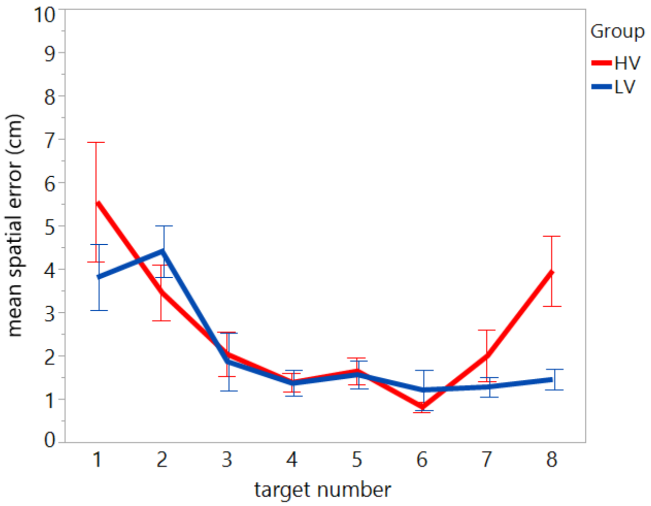

S5 Fig 1. Experiment 1. Analysis of spatial error across consecutive targets at R1 of Block1

We then performed a Bayesian Independent Samples T-Test on the average spatial error in HV and LV groups (the spatial error was averaged across the eight targets at R1 of Block 1 separately for both groups).We tested the hypotheses that H1: spatial error of HV at R1 of Block 1 ≠ spatial error of LV at R1 of Block 1; and H_0: spatial error of HV at R1 of Block 1= spatial error of LV at R1 of Block 1. The prior is described by a Cauchy distribution centred around zero and a default width parameter of 0.707. The analysis resulted in a Bayes Factor, $BF_{01} = 1.340$ (see S5 Table 1 for descriptive). This value of the Bayes Factor indicates that the data are 1.340 times more likely to occur under H_0 compared to H_1. The Bayes Factor of 1.340 provides anecdotal evidence in favour of the null hypothesis (that HV and LV groups are not different).

Group Descriptives

	Group	N	Mean	SD	SE	95% Credible Interval	
						Lower	Upper
Spatial Error	HV	18	2.601	1.180	0.278	2.014	3.188
	LV	18	2.114	0.752	0.177	1.740	2.488

S5 Table 1. Descriptive statistics. Descriptives of the comparison of spatial error in HV and LV groups, at R1 of Block 1, by means of a Bayesian independent t-test.

Experiment 2

The ANOVA with Greenhouse-Geisser correction ($\varepsilon = 0.354$) on the participant's spatial error at R1 of Block 1 revealed a main effect of the Targets, indicating that participants reduced their spatial over the first eight targets ($F (7, 259) = 6.139$, $p<0.0001$, $\eta^2 =0.142$, see S5 Fig 2). The analysis did not show a main effect of Group, ($F(1,38)=0.435$, $p= 0.514$, $\eta^2 = 0.12$; HV group: mean= 2.315, SE= 0.335; LV group: mean= 2.007, SE= 0.326). The interaction between the two factors was significant ($F (7,266) = 4.107$, $p <0.001$, $\eta^2 =0.100$). Post-hoc analyses revealed that R2 of HV group (mean= 7.280, SE= 1.581) was significantly different from that of LV Group (mean= 2.278, SE= 1.541). The higher mean at R2 was due to particularly higher error produced by three participants at Target 2.

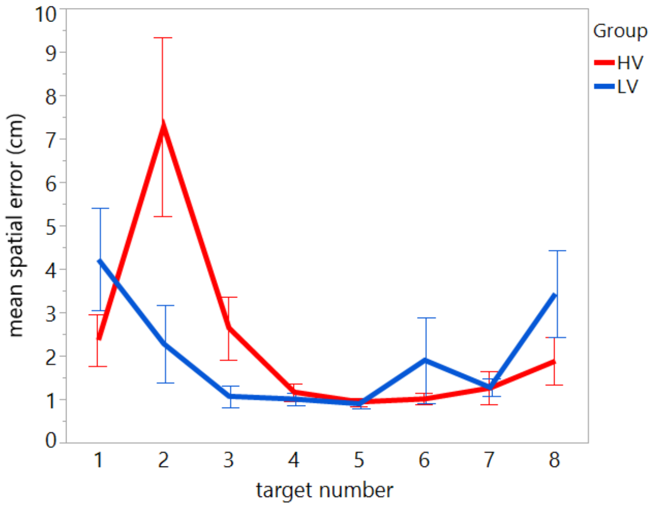

S5 Fig 2. Experiment 2. Analysis of spatial error across consecutive targets at R1 of Block1

A Bayesian Independent Samples T-Test was performed to assess the likelihood of the spatial error in the HV and LV group being equal. The spatial error at all eight targets at R1 of Block 1 was averaged and subjected to comparison. We tested the null hypothesis, H_0: spatial error of HV at R1 of Block 1= spatial error of LV at R1 of Block 1 and the alternate hypothesis, H_1: spatial error of HV at R1 of Block 1 $\neq$ spatial error of LV at R1 of Block 1. The prior is described by a Cauchy distribution centred around zero and a default width parameter of 0.707. The analysis resulted in a Bayes Factor, BF_{01} = 2.698 (see S5 Table 2 for descriptives). This value of the Bayes Factor indicates anecdotal evidence for H_0, which means that the data are 2.698 times more likely to occur under H_0 compared to H_1. The Bayes Factor provides anecdotal evidence in favour of the null hypothesis (that HV and LV groups are not different).

Group Descriptives

	Group	N	Mean	SD	SE	95% Credible Interval	
						Lower	Upper
Spatial Error	HV	19	2.315	1.554	0.357	1.566	3.065
	LV	20	2.007	1.363	0.305	1.369	2.645

S5 Table 2. Descriptive statistics. Descriptives of the comparison of spatial error in HV and LV groups, at R1 of Block 1, by means of a Bayesian independent t-test.

Experiment 3

The ANOVA with Greenhouse-Geisser correction ($\varepsilon = 0.465$) on the participant's spatial error at R1 of Block 1 revealed a main effect of Target as in previous two experiments, indicating that participants reduced their spatial error over the 8 targets ($F (7, 259) = 7.976$, $p<0.0001$, $\eta^2 =0.177$, see S5 Fig 3). The main effect of group (HV group: mean= 1.539, SE= 0.172 and LV group: mean= 1.557, SE= 0.168) was not significant ($F(1,37)=0.005$, $p= 0.941$, $\eta^2 = 0.000$). The interaction between the two factors was also not significant ($F (7, 259) = 0.849$, $p= 0.548$).

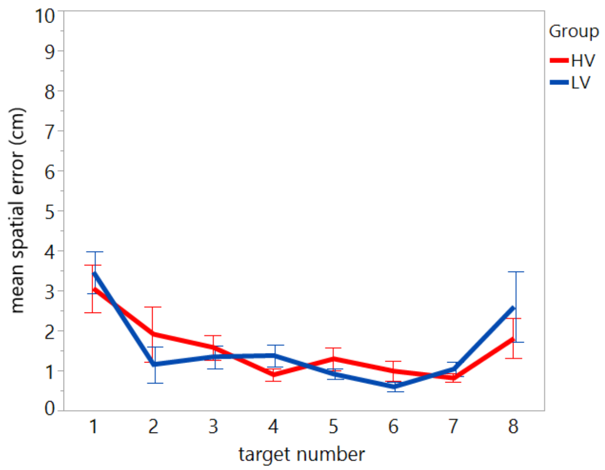

S5 Fig 3. Experiment 3. Analysis of spatial error across consecutive targets at R1 of Block1

A Bayesian Independent Samples T-Test was performed to assess the likelihood of the spatial error in the HV and LV group being equal. The spatial error at all eight targets at R1 of Block 1 was averaged and subjected to comparison. We tested the null hypothesis, H_0: spatial error of HV at R1 of Block 1= spatial error of LV at R1 of Block 1 and the alternate hypothesis, H_1: spatial error of HV at R1 of Block 1 ≠ spatial error of HV at R1 of Block 1. The prior is described by a Cauchy distribution centred around zero and a default width parameter of 0.707. The analysis resulted in a Bayes Factor, BF_{01} = 3.199 (see Table 3 for descriptive). This value of the Bayes Factor indicates moderate evidence for H_0, which means that the data are 3.199 times more likely to occur under H_0 than H_1. The Bayes Factor provides moderate evidence in favour of the null hypothesis, i.e. that HV and LV groups are not different.

Group Descriptives

						95% Credible Interval	
	Group	N	Mean	SD	SE	Lower	Upper
Spatial Error	HV	19	1.539	0.727	0.167	1.189	1.890
	LV	20	1.557	0.770	0.172	1.197	1.917

S5 Table 3. Descriptive statistics. Descriptives of the comparison of spatial error in HV and LV groups, at R1 of Block 1, by means of a Bayesian independent t-test.

The Bayesian analyses in Experiment 1 and 2 provide anecdotal evidence for no difference in spatial error between the two groups and the analysis on Experiment 3 provides moderate evidence for the same. These results along with the repeated measures ANOVA, provide converging evidence that the performance level of the two groups in R1 are not different. As our predictability manipulation only works from the second repetition on as one can only discover

weather their partner is predictable or not through practice, similar performance at R1 in Block 1 suggests that both groups start at the same performance level in all three experiments. In Experiment 1, upon exposure to partner's variability produced in an unpredictable manner, HV group suffers a disadvantage, but only later in the course of training (which was shown in the main analysis with the grand averaged spatial error).

Influence of confederates' 'improvement in performance' on participants' performance

To investigate whether participants' performance was influenced by the confederates' improvements in performance (observed from main effect of Repetition in the confederates' data in all three experiments; see supporting information, S2 File), we analysed whether the correlation between the two actor's spatial errors predicts how much the participant learns. We computed correlations between the participant's and confederate's spatial errors, on a trial by trial basis, for each pair separately. We then performed a group correlation analysis where we correlate r values with the participants' averaged spatial error (Analysis 1) and with the participants' difference in performance between Block 1 and Block 8, indicating the magnitude of participants' learning (Analysis 2).

A significant correlation in Analysis 1, between the r values and the averaged spatial error would indicate that participants' spatial error can be predicted by the degree of correlation between the actors. On the contrary, if the correlations are not significant, this would indicate that there is no significant relationship between the degree of correlation between the actors and the participant's spatial error performance implying that there is no indication that the learners' individual learning profile was dependent on the confederate's performance. Similarly, Analysis

2 would reveal how the degree of correlation between the actors predicts the participants' overall learning performance.

Experiment 1

A Pearson product-moment correlation was computed to determine the relationship between r values (correlations for participant and confederate's spatial error) produced for each pair and the overall spatial error of the corresponding participant in the pair (Analysis 1, see S5 Fig 4). There was no relationship between the variables neither in the HV group (r=0.178, n= 18, p= 0.478) nor the LV group (r= -0.187, n= 18, p= 0.456). A Pearson product-moment correlation was computed to determine the relationship between r values (correlations for participant and confederate's spatial error) produced for each pair and the difference in performance between Block 1 and Block 8 of that participant (Analysis 2). The analysis also did not show any significant relationship between the two variables in HV group (r= 0.192, n= 18, p= 0.435) nor LV group (r= -0.209, n=18, p= 0.403).

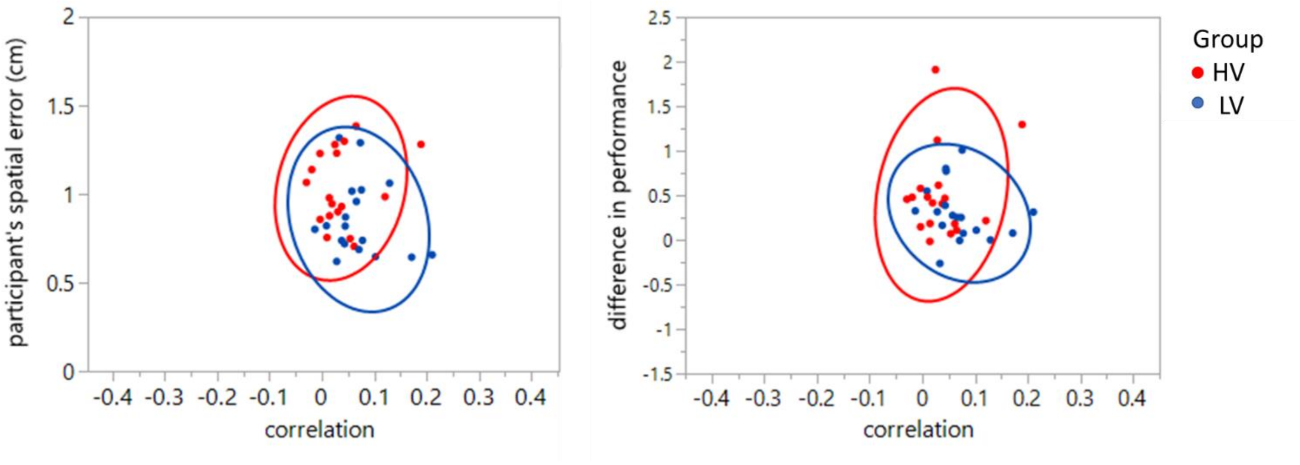

S5 Fig 4. Correlation between inter-personal correlation values and participants' performance in Experiment 1. (A) Analysis 1 (B) Analysis 2

Experiment 2

The Pearson product-moment correlation computed to determine the relationship between r values and the overall spatial error of the participants (Analysis 1, see S5 Fig 5) did not reveal any correlation between the variables neither in the HV group (r= -0.064, n= 20, p= 0.785) nor the LV group (r= -0.340, n= 19, p=0.153). The Pearson product-moment correlation performed on the r values and the difference in performance between Block 1 and Block 8 of the participant (Analysis 2) also did not show any relationship between the two variables neither in HV group (r= -0.019, n= 20, p= 0.934 nor in LV group (r= 0.250, n= 20, p= 0.287).

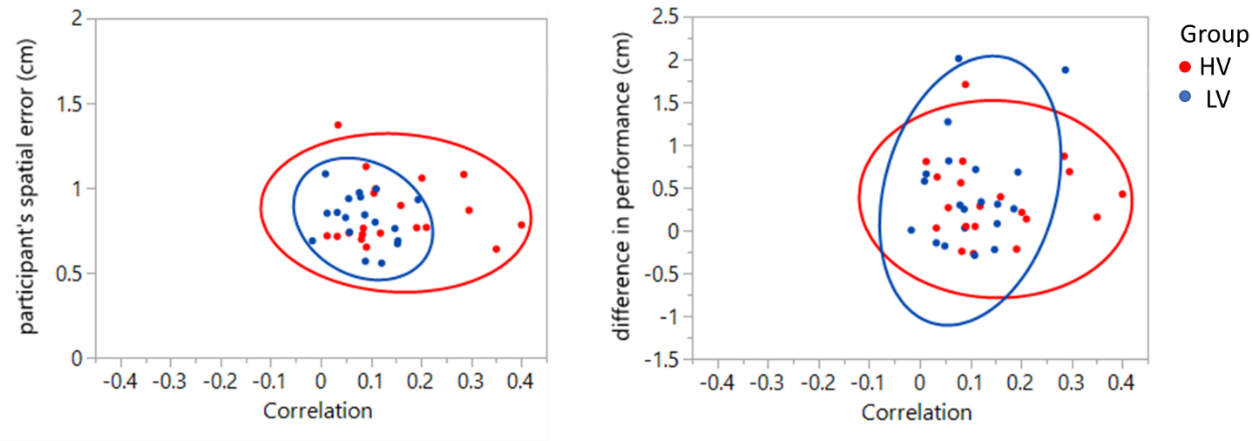

S5 Fig 5. Correlation between inter-personal correlation values and participants' performance in Experiment 2. (A) Analysis 1 (B) Analysis 2

Experiment 3

The Pearson product-moment correlation computed to determine the relationship between r values and the overall spatial error of the participants (see S5 Fig 6) did not reveal any significant relationship between the variables neither in the HV group (r= -0.156, n= 19, p= 0.521) nor in the LV group (r= -0.430, n= 19, p= 0.065). The correlation on the r values and the overall change in

performance between Block 1 and Block 8 of the participant was also not significant neither in HV group (r= 0.275, n= 19, p= 0.253) nor in the LV group (r= 0.256, n= 19, p =0.289).

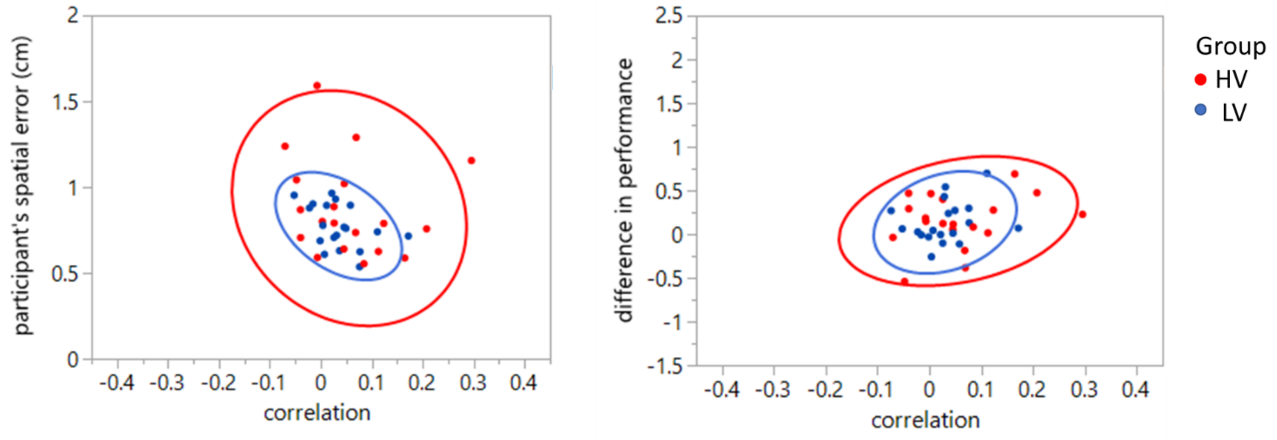

S5 Fig 6. Correlation between inter-personal correlation values and participants' performance in Experiment 3. (A) Analysis 1 (B) Analysis 2

We collapsed the data from all three experiments to test whether the interactors' correlations predicted the participants' performance and learning regardless of the experimental manipulation (see S5 Fig 7). Analysis 1 did not reveal any significant correlation between the inter-actor correlations and the spatial error performance of the participants in HV (r= -0.132, p = 0.329, n= 56) or LV group (r= -0.223, p= 0.098, n= 56). Analysis 2 also did not reveal any significant correlation between the inter-actor correlations and the participants' learning in either group (HV: r= 0.098, p= 0.477, n= 54; LV: r= 0.076, p= 0.583, n= 54).

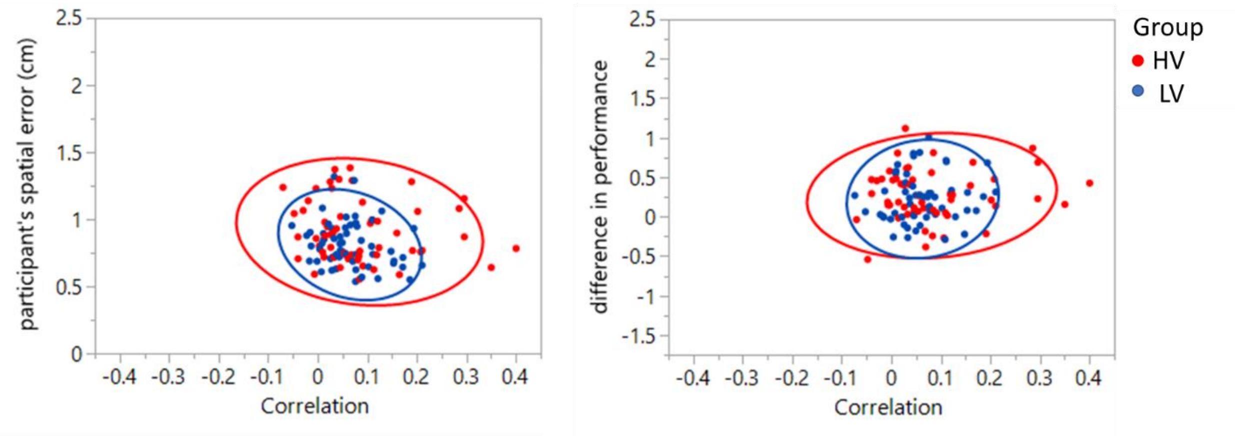

S5 Fig 7. Correlation between inter-personal correlation values and participants' performance from Experiment 1, 2 and 3. (A) Analysis 1 (B) Analysis 2

The results of the correlation analyses did not provide evidence that the degree of correlation between partners' movements (which can be expected given the haptic coupling in our experimental setup) predicts the degree of participants' improvement over time. There is only an indication of the same in Analysis 1 of Experiment 3 conducted on the LV group data (r= -0.430, n= 19, p= 0.065), however, the results were not significant. This seems to indicate that we can separate the influence of the confederate's movements on the learners' movement (which is inevitable provided our experimental set up) from the learners' individual learning profile, which seem to be independent from the confederate's performance.
