# Supplementary material for: How does a partner’s motor variability affect joint action?
Source: PLoS One. 2020 Oct 29;15(10):e0241417. doi: 10.1371/journal.pone.0241417 (PMC7595416; doi:10.1371/journal.pone.0241417)
Supplement: Attachment — Submitted filename: Response to Reviewers.docx [file pone.0241417.s008.docx]

**FW: PLOS ONE Decision: Revision required [PONE-D-20-03780R1] - [EMID:501b7d3d821dc98e]**

Reviewers' comments:

Reviewer's Responses to Questions

**Comments to the Author**

1. If the authors have adequately addressed your comments raised in a previous round of review and you feel that this manuscript is now acceptable for publication, you may indicate that here to bypass the “Comments to the Author” section, enter your conflict of interest statement in the “Confidential to Editor” section, and submit your "Accept" recommendation.

Reviewer #1: All comments have been addressed

Reviewer #2: (No Response)

2. Is the manuscript technically sound, and do the data support the conclusions?

The manuscript must describe a technically sound piece of scientific research with data that supports the conclusions. Experiments must have been conducted rigorously, with appropriate controls, replication, and sample sizes. The conclusions must be drawn appropriately based on the data presented.

Reviewer #1: (No Response)

Reviewer #2: Partly

3. Has the statistical analysis been performed appropriately and rigorously?

Reviewer #1: (No Response)

Reviewer #2: No

4. Have the authors made all data underlying the findings in their manuscript fully available?

The [PLOS Data policy](http://www.plosone.org/static/policies.action#sharing) requires authors to make all data underlying the findings described in their manuscript fully available without restriction, with rare exception (please refer to the Data Availability Statement in the manuscript PDF file). The data should be provided as part of the manuscript or its supporting information or deposited to a public repository. For example, in addition to summary statistics, the data points behind means, medians and variance measures should be available. If there are restrictions on publicly sharing data—e.g. participant privacy or use of data from a third party—those must be specified.

Reviewer #1: (No Response)

Reviewer #2: Yes

5. Is the manuscript presented in an intelligible fashion and written in standard English?

PLOS ONE does not copyedit accepted manuscripts, so the language in submitted articles must be clear, correct, and unambiguous. Any typographical or grammatical errors should be corrected at revision, so please note any specific errors here.

Reviewer #1: (No Response)

Reviewer #2: Yes

6. Review Comments to the Author

Please use the space provided to explain your answers to the questions above. You may also include additional comments for the author, including concerns about dual publication, research ethics, or publication ethics. (Please upload your review as an attachment if it exceeds 20,000 characters)

Reviewer #1: (No Response)

Reviewer #2: I have reviewed the revised manuscript submitted by Sabu et al. I thank the authors for the clarifications and the extensive changes but I feel that there are still 2 main concerns:

1. My concern about the predictability argument does not seem to be resolved. The authors show analysis of the 'first block' (the forces in the first block *has* to be 'unpredictable' by definition because there is no other prior experience from which the participant can predict anything). Yet the differences exist between Exp1 and 2/3 even in this block. I think this *severely* undermines the argument about predictability of forces being the main driving factor.
In their response, the authors argue "We would like to argue that this could be because the pre-exposure to the target locations in the individual phase was enough for the participants to offset the group effect while learning the task together with a partner producing atleast partially predictable movements". I'm not entirely sure what this means. How could the exposure to the target locations in the individual phase (when there were no forces involved) help the learner 'predict' the forces produced by the confederate in Exp 2/3 the first time they experienced these forces? To me, this indicates that some other confound is present in the data

**Reply: We addressed the reviewers concern about the predictability argument by zooming in to the Block 1 analysis which was shown in the previous revision. We now analyse the average spatial error performance of the first repetition (R1) of the first block - which includes 8 different target locations. This represents the first instance of participants’ performance of a sequence (8 targets) in the presence of force perturbations. Since the first repetition of the first block is unpredictable regardless of the experimental condition, one would expect no difference in performance for all participants in both groups and across the three experiments. We subjected the participants’ spatial error to a 2 x 8 mixed ANOVA with Group (2) as the between-subjects factor and Target Number (8) as the within-subjects factor. These analyses are included in the supporting information, S5 File.**

**Experiment 1: The ANOVA, with Greenhouse-Geisser correction (ε = 0.435), on the participant’s spatial error at R1 of Block 1 revealed a main effect of the Targets, indicating that participants reduced their spatial error across consecutive targets (F (7, 238) = 10.047, p<0.0001, η^2^ =0.228, see S5 Fig 1). The main effect of Group (HV group: mean= 2.601, SE= 0.233 and LV group: mean= 2.114, SE= 0.233) failed to reach a significance (F(1,34)= 2.183, p= 0.149, η^2^ =0.060). The interaction between the two factors was also not significant (F (7,238) = 1.901, p= 0.070, η^2^ =0.053).**

**S5 Fig 1. Experiment 1. Analysis of spatial error across consecutive targets at R1 of Block1**

**We then performed a Bayesian Independent Samples T-Test on the average spatial error in HV and LV groups (the spatial error was averaged across the eight targets at R1 of Block 1 separately for both groups).We tested the hypotheses that H1: spatial error of HV at R1 of Block 1 ≠ spatial error of LV at R1 of Block 1; and H_0_: spatial error of HV at R1 of Block 1= spatial error of LV at R1 of Block 1. The prior is described by a Cauchy distribution centred around zero and a default width parameter of 0.707. The analysis resulted in a Bayes Factor, BF_01_ = 1.340 (see S5 Table 1 for descriptive). This value of the Bayes Factor indicates that the data are 1.340 times more likely to occur under H_0_ compared to H_1_. The Bayes Factor of 1.340 provides anecdotal evidence in favour of the null hypothesis (that HV and LV groups are not different).**

| **Group Descriptives** | | | | | | | | | | | | | | | |
| --- | --- | --- | --- | --- | --- | --- | --- | --- | --- | --- | --- | --- | --- | --- | --- |
|  | | | | | | | | | | | | **95% Credible Interval** | | | |
|  | | **Group** | | **N** | | **Mean** | | **SD** | | **SE** | | **Lower** | | **Upper** | |
| Spatial Error |  | HV |  | 18 |  | 2.601 |  | 1.180 |  | 0.278 |  | 2.014 |  | 3.188 |  |
|  |  | LV |  | 18 |  | 2.114 |  | 0.752 |  | 0.177 |  | 1.740 |  | 2.488 |  |
|  | | | | | | | | | | | | | | | |

**S5 Table 1. Descriptive statistics: Descriptives of the comparison of spatial error in HV and LV groups, at R1 of Block 1, by means of a Bayesian independent t-test.**

**Experiment 2: The ANOVA with Greenhouse-Geisser correction (ε = 0.354) on the participant’s spatial error at R1 of Block 1 revealed a main effect of the Targets, indicating that participants reduced their spatial over the first eight targets (F (7, 259) = 6.139, p<0.0001, η^2^ =0.142, see S5 Fig 2). The analysis did not show a main effect of Group, (F(1,38)=0.435, p= 0.514, η^2^ = 0.12; HV group: mean= 2.315, SE= 0.335; LV group: mean= 2.007, SE= 0.326). The interaction between the two factors was significant (F (7,266) = 4.107, p <0.001, η^2^ =0.100). Post-hoc analyses revealed that R2 of HV group (mean= 7.280, SE= 1.581) was significantly different from that of LV Group (mean= 2.278, SE= 1.541). The higher mean at R2 was due to particularly higher error produced by three participants at Target 2.**

**S5 Fig 2. Experiment 2. Analysis of spatial error across consecutive targets at R1 of Block1**

**A Bayesian Independent Samples T-Test was performed to assess the likelihood of the spatial error in the HV and LV group being equal. The spatial error at all eight targets at R1 of Block 1 was averaged and subjected to comparison. We tested the null hypothesis, H_0_: spatial error of HV at R1 of Block 1= spatial error of LV at R1 of Block 1 and the alternate hypothesis, H_1_: spatial error of HV at R1 of Block 1 ≠ spatial error of LV at R1 of Block 1. The prior is described by a Cauchy distribution centred around zero and a default width parameter of 0.707. The analysis resulted in a Bayes Factor, BF_01_ = 2.698 (see S5 Table 2 for descriptives). This value of the Bayes Factor indicates anecdotal evidence for H_0_, which means that the data are 2.698 times more likely to occur under H_0_ compared to H_1_. The Bayes Factor provides anecdotal evidence in favour of the null hypothesis (that HV and LV groups are not different).**

| **Group Descriptives** | | | | | | | | | | | | | | | |
| --- | --- | --- | --- | --- | --- | --- | --- | --- | --- | --- | --- | --- | --- | --- | --- |
|  | | | | | | | | | | | | **95% Credible Interval** | | | |
|  | | **Group** | | **N** | | **Mean** | | **SD** | | **SE** | | **Lower** | | **Upper** | |
| Spatial Error |  | HV |  | 19 |  | 2.315 |  | 1.554 |  | 0.357 |  | 1.566 |  | 3.065 |  |
|  |  | LV |  | 20 |  | 2.007 |  | 1.363 |  | 0.305 |  | 1.369 |  | 2.645 |  |
|  | | | | | | | | | | | | | | | |

**S5 Table 2. Descriptive statistics: Descriptives of the comparison of spatial error in HV and LV groups, at R1 of Block 1, by means of a Bayesian independent t-test.**

**Experiment 3: The ANOVA with Greenhouse-Geisser correction (ε = 0.465) on the participant’s spatial error at R1 of Block 1 revealed a main effect of Target as in previous two experiments, indicating that participants reduced their spatial error over the 8 targets (F (7, 259) = 7.976, p<0.0001, η^2^ =0.177, see S5 Fig 3). The main effect of group (HV group: mean= 1.539, SE= 0.172 and LV group: mean= 1.557, SE= 0.168) was not significant (F(1,37)=0.005, p= 0.941,η^2^ = 0.000). The interaction between the two factors was also not significant (F (7, 259) = 0.849, p= 0.548).**

**S5 Fig 3. Experiment 3. Analysis of spatial error across consecutive targets at R1 of Block1**

**A Bayesian Independent Samples T-Test was performed to assess the likelihood of the spatial error in the HV and LV group being equal. The spatial error at all eight targets at R1 of Block 1 was averaged and subjected to comparison. We tested the null hypothesis, H_0_: spatial error of HV at R1 of Block 1= spatial error of LV at R1 of Block 1 and the alternate hypothesis, H_1_: spatial error of HV at R1 of Block 1 ≠ spatial error of HV at R1 of Block 1. The prior is described by a Cauchy distribution centred around zero and a default width parameter of 0.707. The analysis resulted in a Bayes Factor, BF_01_ = 3.199 (see Table 3 for descriptive). This value of the Bayes Factor indicates moderate evidence for H_0_, which means that the data are 3.199 times more likely to occur under H_0_ than H_1_. The Bayes Factor provides moderate evidence in favour of the null hypothesis, i.e. that HV and LV groups are not different.**

| **Group Descriptives** | | | | | | | | | | | | | | | |
| --- | --- | --- | --- | --- | --- | --- | --- | --- | --- | --- | --- | --- | --- | --- | --- |
|  | | | | | | | | | | | | **95% Credible Interval** | | | |
|  | | **Group** | | **N** | | **Mean** | | **SD** | | **SE** | | **Lower** | | **Upper** | |
| Spatial Error |  | HV |  | 19 |  | 1.539 |  | 0.727 |  | 0.167 |  | 1.189 |  | 1.890 |  |
|  |  | LV |  | 20 |  | 1.557 |  | 0.770 |  | 0.172 |  | 1.197 |  | 1.917 |  |
|  | | | | | | | | | | | | | | | |

**S5 Table 3. Descriptive statistics: Descriptives of the comparison of spatial error in HV and LV groups, at R1 of Block 1, by means of a Bayesian independent t-test.**

**The Bayesian analyses in Experiment 1 and 2 provide anecdotal evidence for no difference in spatial error between the two groups and the analysis on Experiment 3 provides moderate evidence for the same. These results along with the repeated measures ANOVA, provide converging evidence that the performance level of the two groups in R1 are not different. As our predictability manipulation only works from the second repetition on as one can only discover weather their partner is predictable or not through practice, similar performance at R1 in Block 1 suggests that both groups start at the same performance level in all three experiments. In Experiment 1, upon exposure to partner’s variability produced in an unpredictable manner, HV group suffers a disadvantage, but only later in the course of training (which was shown in the main analysis with the grand averaged spatial error).**

Relatedly, the finding that the confederate also showed 'improvements in performance' and differences between conditions is worrying because it creates a confound in interpretation of the learner's performance. Because the confederate is haptically coupled to the learner the confederate's errors and the related forces they exert also influence the learner's spatial error. (i.e. imagine if the learner had the intent to make the exact same motion each time - the movement of the confederate would influence how the spatial error of the learner). This is also why I disagree with the characterization that spatial error is an 'individual' performance metric in Ln 172 - when the forces are coupled spatial error is no longer an individual measure of performance.

**Reply:** **To investigate whether participants’ performance was influenced by the confederate’s improvements in performance, we analysed whether the correlation between the two actor’s spatial errors predicts how much the participant learns. We computed correlations between the participant’s and confederate’s spatial errors, on a trial by trial basis, for each pair separately. We then performed a group correlation analysis where we correlate r values with the participants’ averaged spatial error (Analysis 1) and with the participants’ difference in performance between Block 1 and Block 8, indicating the magnitude of participants’ learning (Analysis 2).**

**A significant correlation in Analysis 1, between the r values and the averaged spatial error would indicate that participants’ spatial error can be predicted by the degree of correlation between the actors. On the contrary, if the correlations are not significant, this would indicate that there is no significant relationship between the degree of correlation between the actors and the participant’s spatial error performance implying that there is no indication that the learners’ individual learning profile was dependent on the confederate’s performance. Similarly, Analysis 2 would reveal how the degree of correlation between the actors predicts the participants’ overall learning performance. These analyses will be included in the supporting information, S5 File.**

**Experiment 1: A Pearson product-moment correlation was computed to determine the relationship between r values (correlations for participant and confederate’s spatial error) produced for each pair and the overall spatial error of the corresponding participant in the pair (Analysis 1, see S5 Fig 4). There was no relationship between the variables neither in the HV group (r=0.178, n= 18, p= 0.478) nor the LV group (r= -0.187, n= 18, p= 0.456). A Pearson product-moment correlation was computed to determine the relationship between r values (correlations for participant and confederate’s spatial error) produced for each pair and the difference in performance between Block 1 and Block 8 of that participant (Analysis 2). The analysis also did not show any significant relationship between the two variables in HV group (r= 0.192, n= 18, p= 0.435) nor LV group (r= -0.209, n=18, p= 0.403).**

**S5 Fig 4. Correlation between inter-personal correlation values and participants’ performance in Experiment 1. (A) Analysis 1 (B) Analysis 2**

**Experiment 2: The Pearson product-moment correlation computed to determine the relationship between r values and the overall spatial error of the participants (Analysis 1, see S5 Fig 5) did not reveal any correlation between the variables neither in the HV group (r= -0.064, n= 20, p= 0.785) nor the LV group (r= -0.340, n= 19, p=0.153). The Pearson product-moment correlation performed on the r values and the difference in performance between Block 1 and Block 8 of the participant (Analysis 2) also did not show any relationship between the two variables neither in HV group (r= -0.019, n= 20, p= 0.934 nor in LV group (r= 0.250, n= 20, p= 0.287).**

**S5 Fig 5. Correlation between inter-personal correlation values and participants’ performance in Experiment 2. (A) Analysis 1 (B) Analysis 2**

**Experiment 3: The Pearson product-moment correlation computed to determine the relationship between r values and the overall spatial error of the participants (see S5 Fig 6) did not reveal any significant relationship between the variables neither in the HV group (r= -0.156, n= 19, p= 0.521) nor in the LV group (r= -0.430, n= 19, p= 0.065). The correlation on the r values and the overall change in performance between Block 1 and Block 8 of the participant was also not significant neither in HV group (r= 0.275, n= 19, p= 0.253) nor in the LV group (r= 0.256, n= 19, p =0.289).**

**S5 Fig 6. Correlation between inter-personal correlation values and participants’ performance in Experiment 3. (A) Analysis 1 (B) Analysis 2**

**We collapsed the data from all three experiments to test whether the interactors’ correlations predicted the participants’ performance and learning regardless of the experimental manipulation (see S5 Fig 7). Analysis 1 did not reveal any significant correlation between the inter-actor correlations and the spatial error performance of the participants in HV (r= -0.132, p = 0.329, n= 56) or LV group (r= -0.223, p= 0.098, n= 56). Analysis 2 also did not reveal any significant correlation between the inter-actor correlations and the participants’ learning in either group (HV: r= 0.098, p= 0.477, n= 54; LV: r= 0.076, p= 0.583, n= 54).**

**S5 Fig 7. Correlation between inter-personal correlation values and participants’ performance from Experiment 1, 2 and 3. (A) Analysis 1 (B) Analysis 2**

**The results of the correlation analyses did not provide evidence that the degree of correlation between partners’ movements (which can be expected given the haptic coupling in our experimental setup) predicts the degree of participants’ improvement over time. There is only an indication of the same in Analysis 1 of Experiment 3 conducted on the LV group data (r= -0.430, n= 19, p= 0.065), however, the results were not significant. This seems to indicate that we can separate the influence of the confederate’s movements on the learners’ movement (which is inevitable provided our experimental set up) from the learners’ individual learning profile, which seem to be independent from the confederate’s performance.**

2. Moreover, related to my concern about theoretical rationale, after reading the response from the authors which provided clarification, I am still unsure how this paper has anything to say about 'learning' in the traditional sense of the word (see performance-learning distinction - see for e.g. Salmoni, Schmidt & Walter, 1984). What the paper measures is 'performance' and its change over time. However, since there is no 'common condition' under which the groups are tested (e.g., a test where all groups switched to the same haptic condition), I believe no inferences about learning (which reflects the underlying change in motor skill) can be made from the data. So it is still not clear to me that the framing of the intro and discussion in terms of Wu et al. and learning/exploration is appropriate here (I think the framing in terms of joint action and how someone regulates their variability in response to a partner's variability is OK)

**Reply: We agree with the reviewer that having a test phase following the training phase would have allowed us to measure how the training contributes to long term retention of the learned skill or the transfer of the learned skill to a different movement context. We acknowledge the lack of a test phase as a limitation of our paradigm.**

**However, we would like to bring to notice that while testing individuals on a new movement condition would have broadened our understanding on the degree of learning occurred, learning could be captured through other means as well. Motor learning and adaptation does occur not only when a previously learned pattern of muscle activations facilitates acquisition of an entirely new pattern of activations, but rather it can also consist in acquiring a new mapping between the learned movements and the desired goal- importantly and relatedly to the current task, a similar but novel movement sequence. It is also important to note that consolidation related to learning is not just a time-dependent process, but it can also happen through reaching a performance saturation within a training session (Krakuer, 2009). Previous research has demonstrated that saturation within a training session is sufficient for consolidation of learning (Krakuer 2009). In addition, there is empirical evidence showing that short-term motor learning can be captured within timescales of even minutes (Smith, Ghazizadeh & Shadmehr, 2006) and also that learning can be captured without introducing catch trials or test phase but by analysing the trial-by-trial change in motor behaviour (Thoroughman and Shadmehr, 2000; Donchin, Francis & Shadmehr, 2003).**

**In our study, every individual performs 640 trials of movement trajectories in Experiment 1 and 320 trials in Experiment 2 and 3 each. This gives our participants sufficient time to reach an asymptote or saturation and this can be observed in our analyses of the spatial error. Additionally, our learning task is not measuring performance over repetitions of a single movement sequence. It is a block design were each group performs 8 different movement sequences - each sequence consisting of 8 trajectories. Each of the block onsets is preceded by a slight delay. If there was no learning or no transfer of previously learned skills to different movement sequences (in our case, different Blocks), participants would have returned to their initial performance level at the beginning of every Block. This is not the case in our study. Moreover, taking together the analysis of the spatial error submitted in the manuscript and the movement time (*see Reply to Pt. 1 under Minor*), it is clear that there is an offset of speed-accuracy trade off in performance - which is a characteristic of skill learning (Willingham,1998; Sanes et al. 1990; Shmuleof et al, 2011).**

**Thus, while we agree that having a common test-phase for both groups would have allowed us to draw conclusions about the effect of the variability manipulation in long term retention of the learned skill, we would like to argue that our paradigm still reflects learning, which is manifested by an underlying change in the motor skill over different movement patterns.**

Minor:

1. It is not specified what the movement times are for these movements (only the cueing time of 1000ms is mentioned). Also, I was referring to the speed-accuracy trade-off for movement time (i.e. Fitts' Law). Movement times here are controlled by the participant and not by the script.

**Reply: Actors in our experiments were instructed to synchronize with an isochronous sequence of tones that dictated moving within the 1000 ms cue period (this has been clarified under the Experimental Timeline of each experiment in the manuscript). Thus, a speed-accuracy trade-off seems unlikely in the present task. Nevertheless, we performed additional analyses on the movement times of the participants. We here report the analysis of participant’s movement times (defined as the time from target to target). Movement time was subjected to a repeated measures ANOVA with Repetitions (10 or 5) as within-subject factor and Group (2) as between-subject factor. The analysis reveals that in all the three experiments, participants show a main effect of repetition, with the movement time at R1 always higher than the final repetition. Taking these results, together with the reduction of spatial error observed in the main analysis of spatial error (submitted in the manuscript) it is evident that, while participants learn to get more accurate in their movements, they also get faster in all three experiments. If there was a speed-accuracy trade-off, the change in movement time and spatial error over repetitions should have followed opposite trends, which is not what we observe in our data. The analysis on the movement times are as follows and these will be included in the supporting information, S4 File.**

**Experiment 1 : “2 x 10 The ANOVA on the participant’s movement time revealed a main effect of the repetitions, indicating that participants in both groups reduced their movement time over repetitions (F (9,306) = 6.612, p<0.0001, η^2^ = 0.163, see S4 Fig 1). Post hoc revealed that the first repetition R1 was significantly different from all other repetitions (mean= 0.842, SE= 0.004, all *p*s< 0.005)The main effect of group (HV group: mean=0.832, SE=0.006 and LV group: mean= 0.829, SE= 0.006) failed to reach a significance (F(1,34)= 0.103, p= 0.7495, η^2^ = 0.003. Even though the interaction between the two factors was significant (F (9, 306) = 2.601, p= 0.007, η^2^ = 0.071), post-hoc analysis did not reveal any significant differences (all *p*s >0.1).”**

**S4 Fig 1. Analysis of participants’ movement time in Experiment 1.**

**Experiment 2 : “The 2 x 5 ANOVA on the participant’s movement time revealed a main effect of Repetition, indicating that participants in both groups showed reduction of movement time across repetitions (F (4,152) = 31.447, p<0.0001, η^2^ =0.453, see S4 Fig 2). Post-hoc analyses revealed that R1 (mean= 0.827, SE=0.005) was significantly higher than all other repetitions (all *p*s<0.0001).The main effect of group (HV group: mean=0.812, SE=0.006 and LV group: mean= 0.806, SE= 0.006) was not significant (F (1,38) = 0.437, p= 0.513, η^2^ =0.011). The interaction between the two factors was significant (F (4,152) = 5.838, p< 0.001, η^2^ = 0.133). Post-hoc revealed that the HV group had a higher movement time at R1 (mean= 0.837, SE= 0.007) compared to the LV group (mean= 0.816, SE= 0.007).”**

**S4 Fig 2. Analysis of participants’ movement time in Experiment 2.**

**Experiment 3: “The 2 x 5 ANOVA on the participant’s movement time revealed a main effect of Repetition, indicating that participants in both groups showed reduction of movement time over repetition (F (4,148) =7.630, p<0.0001, η^2^ =0.171, see S4 Fig 3). Post-hoc revealed that movement time at R1 (mean= 0.826, SE= 0.004) was significantly higher compared to R2-R4 (all *p*s <0.006). The main effect of group (HV group: mean= 0.818, SE=0.008 and LV group: mean= 0.820, SE= 0.008) did not reach a significance (F(1,37)= 0.099, p= 0.755, η^2^ =0.003). The interaction between the two factors was significant (F (4,148) = 2.665, p= 0.034, η^2^ = 0.067). However, post-hoc tests did not reach a significance (all *p*s>0.16).”**

**S4 Fig 3. Analysis of participants’ movement time in Experiment 3.**

2. Ln 332-333: I think the outlier stats need to go under Ln 345 where the outlier criteria is mentioned

**Reply: Done**

3. Ln 801 - if the authors wish to compare the magnitude of the horizontal to the vertical variability, then it should be normalized to the amplitude of the motion (i.e. a higher amplitude motion along the horizontal will give rise to higher variability along that dimension because of signal dep noise)

**Reply: We agree with the reviewers point that there is a potential confound regarding the movements required from the actors as the target grid is wider on the horizontal dimension than the vertical dimension. However, this is constant across sequences and experiments. Regardless, we would like to show the analysis on the movement variability, with the variability on the horizontal and vertical dimension normalized with the distance travelled by the individual at every trajectory. Report of the analysis reads as following in the results section of Experiment 1, 2 and 3 in the manuscript:**

**Experiment 1: “The mixed ANOVA revealed a main effect of Repetition with Greenhouse-Geisser correction (ε =0.721), indicating that participants in both groups reduced their variability over time (F (9, 306) = 7.926, p<0.0001, η^2^ = 0.189, see Fig 5A). Post-hoc analyses revealed that variability at first repetition, R1 (mean=0.120, SE= 0.007) was significantly higher compared to other repetitions (all ps<0.005). The main effect of axis-dimension was also significant (F(1,34)=22.885, p<0.0001, η^2^ =0.402) with the variability on the horizontal dimension being larger (mean=0.091, SE= 0.004) than the variability on the vertical dimension (mean= 0.074, SE= 0.004). The analysis also showed a significant main effect of group (F(1,34)= 11.667, p=0.002, η^2^ = 0.255), indicating that the HV group participants had a higher movement variability (mean= 0.095, SE=0.005) compared to the LV group (mean= 0.069, SE=0.005). The interaction between repetition and group (F (9,306)= 2.443, p= 0.011, η^2^ =0.067) was significant with Greenhouse-Geisser correction (ε =0.509), with the largest mean difference driven by the higher variability of HV group participants at R1 (mean=0.148 , SE=0.011) compared to LV group (mean= 0.093, SE= 0.011). This was confirmed by post-hoc analysis (p=0.001). This indicates a faster reduction of variability by HV group compared to LV group. All other interactions were non-significant (ps >0.06; see Supporting Information S1 File).”**

**Fig 5. Results of Experiment 1. (A) Normalized variability of participant’s movement in the horizontal and vertical dimension.**

**Experiment 2: “****The mixed ANOVA revealed a main effect of Repetition with Greenhouse-Geisser correction (ε =0.705), indicating that participants in both groups reduced their variability over time (F(4,152)=13.384, p<0.0001, η^2^ =0.260, see Fig 7A). Post-hoc analysis revealed that the variability at first repetition, R1 (mean= 0.092, SE=0.008) was significantly higher compared to other repetitions (all *p*s<0.01) and R5 was also significantly different from all other repetitions(mean= 0.045, SE= 0.004, all *p*s<0.025). The main effect of axis-dimension was also significant (F(1,38)= 55.940, p<0.0001, η^2^ =0.595) with the variability on the horizontal dimension being larger (mean=0.075, SE=0.005) than the variability on the vertical dimension (mean=0.054, SE=0.04).The main effect of group did not reach a significance (F(1,38)= 1.289, p= 0.263, η^2^ =0.033) (HV group: mean=0.060, SE= 0.006; LV Group: mean=0.069, SE= 0.006). The interaction between repetition and axis-dimension (F(4,152)=6.640, p<0.0001, η^2^ = 0.149) was found to be significant with a Greenhouse-Geisser correction (ε =0.668). Post-hoc revealed that the variability on the horizontal dimension was significantly higher than the vertical dimension in all 5 repetitions (all *p*s*<0.005*)*.* There was also a significant interaction between the group and axis-dimension (F(1, 38)= 10.544, p<0.001, η^2^ =0.595) However, post-hoc analyses did not reveal any significant difference (all *p*s>0.07). All other interactions were non-significant (*p*s > 0.86; see Supporting Information S1 File).”**

**Fig 7. Results of Experiment 2. (A) Normalized Variability of participant’s movement in the horizontal and vertical dimension.**

**Experiment 3: “****The mixed ANOVA revealed a main effect of Repetition, indicating that participants in both groups reduced their variability over time (F(4,148)=5.992, p<0.0001, η^2^ = 0.139, see Fig 8A). Post-hoc analysis revealed that the variability at first repetition, R1 (mean=0.076, SE= 0.005) was significantly higher compared to other repetitions (all *p*s<0.01). The main effect of axis-dimension was also significant (F(1,37)=26.637, p<0.0001, η^2^ =0.419) with the variability on the horizontal dimension being larger (mean= 0.066, SE= 0.004) than the variability on the vertical dimension (mean= 0.055, SE= 0.004). The main effect of group did not reach a significance (F(1,37) = 1.975, p= 0.168, η^2^ =0.051; HV group: mean=0.066, SE= 0.005; LV group: mean=0.056, SE= 0.005). None of the interactions were significant (all *p*s >0.14; see Supporting Information S1 File).”**

**Fig 8. Results of Experiment 3. (A) Normalized variability of participant’s movement in the horizontal and vertical dimension.**

4. Ln 536 - I'm not sure if this is what the authors intended but the spatial error (what I Had referred to as bivariate error) is not the average of the x- and y- components (since it also has take into account the correlation between them in diagonal movements for example).See for example (Hancock, Butler, and Fischman (1995))

**Reply: We apologize for the wrong phrasing. The variability was not calculated as the average deviations of the spatial error, rather it was calculated as the average deviations of the x and y *coordinates* separately. The text has been edited accordingly.**

5. For the Bayesian test, please indicate what prior was used. Also in Table 1, the N column needs to be made wider so that 20 is in the same row

**Reply: Done**

6. For the correlations, there are both within- and between-subject data pooled to make a correlation- this inference can be erroneous (see for eg. Simpson's paradox). Either this correlation needs to be done at an individual level and then averaged or the nested nature of this data needs to be accounted for.

Also this is a correlation between two continuous variables - so I don't fully understand why the LV group cannot be plotted (yes, their range will be smaller and this can be acknowledged in the Discussion, but it would be helpful to see the correlation plots anyway)

**Reply: To avoid the Simpson’s paradox in our analysis, we subjected the force and spatial error measure to a repeated-measures correlation (rmcorr), where the inter-individual variability is considered while computing the correlation values (Bakdash & Marusich, 2017). Furthermore, we separated the repetitions and look only at the first and the last repetitions in the current analysis, which provides an estimate of how participants learned to adapt to force perturbations over the course of the experiment. We hope this will address the reviewer’s concern about the analysis. We would also like to bring to the notice that the unusual higher degree of freedom reported is because rmcorr uses repeated measures (Bakdash & Marusich, 2017). The new results do not seem to change the pattern reported in the manuscript, except that Experiment 2 analysis currently gives more evidence for adaptation to larger forces under conditions of complete predictability.**

**Report of the new analyses on the HV group is included in the manuscript under the result sections of Experiment 1, 2 and 3 and it reads as following:**

**Experiment 1:** **“A repeated measures correlation (rmcorr) was computed to determine the relationship between force perturbation and the spatial error in the HV group participants. The correlation was done on the first (R1) and last repetitions (R10) separately. The analysis revealed a significant correlation between the force perturbation experienced and the spatial error at R1 (r_rm_(364)=0.290, p < 0.001, 95% CI= 0.1935913 0.3819815) and as well as at R10 (r_rm_(414)=0.126, p =0.009, 95% CI= 0.03092686 0.2206055). A scatter plot (see Fig 5C) summarizes the results. The results show that participants made more errors when they experienced larger force perturbations.”**

R10

R1

**Fig 5. Results of Experiment 1. (C) Repeated measures correlational analysis between the force perturbation experienced and the spatial error of the participants in the HV group, for R1 and R10 separately.** **Each participant's data and corresponding rmcorr fit lines are shown in different colors.**

**Experiment 2: “****A repeated measures correlation (rmcorr) was computed to determine the relationship between force perturbation and spatial error in the HV group for R1 and R5 separately. The analysis revealed a significant negative correlation between the force perturbation experienced and the spatial error at R1 r_rm_ (437)= -0.154, p =0.001; 95% CI= 0.007834378, 0.1892928) and at R5 (r_rm_ (447)= -0.135, DF =447, p =0.004; 95% CI= -0.2254212 -0.04330164). A scatter plot summarizes the results (see Fig 7C). The results indicate that in the HV group, participants achieved higher accuracy when encountering larger perturbations under conditions of complete predictability, indicating that they learned to adapt to larger forces by becoming more resilient.”**

R1

R5

**Fig 7. Results of Experiment 2. (C) Repeated measures correlational analysis between the force perturbation experienced and the spatial error of the participants in the HV group, for R1 and R5 separately. Each participant's data and corresponding rmcorr fit lines are shown in different colors.**

**Experiment 3: “****A repeated measures correlation (rmcorr) was computed for R1 and R5 separately, to determine the relationship between the force perturbation experienced and the spatial error in the HV group. The analysis revealed a significant correlation between the force perturbation and the spatial error at R1 (r_rm_(415)= 0.131, p =0.007, 95% CI= 0.0355664 0.2247967). However, there was no correlation observed at R5 (r_rm_(385)= 0.067, p = 0.181, 95% CI= -0.03217353 0.166803) suggesting that participants learned to adapt to larger forces as in Experiment 2, over the course of training. A scatter plot (see Fig 8C) summarizes the results.”**

R5

R1

**Fig 8. Results of Experiment 3. (C) Repeated measures correlational analysis between the force perturbation experienced and the spatial error of the participants in the HV group, for R1 and R5 separately. Each participant's data and corresponding rmcorr fit lines are shown in different colors.**

**Report of the rmcorr analyses on the LV group is included in the Supporting Information, S3 File and it reads as following:**

**Experiment 1: “A repeated measures correlation (rmcorr) was computed for R1 and R10 separately, to determine the relationship between the force perturbation experienced and the spatial error in the LV group. The correlation analysis did not reveal any significant correlations neither at R1 (r_rm_(142)=-0.082, p =0.328, 95% CI= -0.2434504 0.083848) nor at R10 (r_rm_(179)=0.019, p =0.794, 95% CI= -0.127582 0.1656539). A scatter plot (see S3 Fig 1) summarizes the results.”**

R1

R10

**S3 Fig 1. Results of Experiment 1. Repeated measures correlational analysis between the force perturbation experienced and the spatial error of the participants in the LV group, for R1 and R10 separately. Each participant's data and corresponding rmcorr fit lines are shown in different colors.**

**Experiment 2: “A repeated measures correlation (rmcorr) was computed for R1 and R5 separately, to determine the relationship between the force perturbation experienced and the spatial error in the LV group. The correlation analysis did not reveal any significant correlations neither at R1 (r_rm_(59)=0.085, p =0.510, 95% CI= -0.1740236, 0.3346091) nor at R10 (r_rm_(59)=0.039, p =0.761, 95% CI= -0.2186034, 0.2928067). A scatter plot (see S3 Fig 2) summarizes the results.”**

R5

R1

**S3 Fig 2. Results of Experiment 2. Repeated measures correlational analysis between the force perturbation experienced and the spatial error of the participants in the LV group, for R1 and R 5 separately. Each participant's data and corresponding rmcorr fit lines are shown in different colors.**

**Experiment 3: “A repeated measures correlation (rmcorr) was computed for R1 and R5 separately, to determine the relationship between the force perturbation experienced and the spatial error in the LV group. The correlation analysis revealed a significant positive correlation at R1 (r_rm_(99)= 0.217, p =0.029; 95% CI= 0.02048916, 0.3974088) and no correlation at R5 (0.029, p = 0.770; 95% CI= -0.1690251, 0.2254713). A scatter plot (see S3 Fig 3) summarizes the results.”**

R5

R1

**S3 Fig 3. Results of Experiment 3. Repeated measures correlational analysis between the force perturbation experienced and the spatial error of the participants in the LV group, for R1 and R5 separately. Each participant's data and corresponding rmcorr fit lines are shown in different colors.**

7. PLOS authors have the option to publish the peer review history of their article ([what does this mean?](https://journals.plos.org/plosone/s/editorial-and-peer-review-process#loc-peer-review-history)). If published, this will include your full peer review and any attached files.


If you choose “no”, your identity will remain anonymous but your review may still be made public.
**Do you want your identity to be public for this peer review?** For information about this choice, including consent withdrawal, please see our [Privacy Policy](https://www.plos.org/privacy-policy).

Reviewer #1: No

Reviewer #2: No

[NOTE: If reviewer comments were submitted as an attachment file, they will be attached to this email and accessible via the submission site. Please log into your account, locate the manuscript record, and check for the action link "View Attachments". If this link does not appear, there are no attachment files.]

While revising your submission, please upload your figure files to the Preflight Analysis and Conversion Engine (PACE) digital diagnostic tool, <https://pacev2.apexcovantage.com/>. PACE helps ensure that figures meet PLOS requirements. To use PACE, you must first register as a user. Registration is free. Then, login and navigate to the UPLOAD tab, where you will find detailed instructions on how to use the tool. If you encounter any issues or have any questions when using PACE, please email PLOS at [figures@plos.org](mailto:figures@plos.org). Please note that Supporting Information files do not need this step.

*In compliance with data protection regulations, you may request that we remove your personal registration details at any time.* [*(Remove my information/details)*](https://www.editorialmanager.com/pone/login.asp?a=r)*. Please contact the publication office if you have any questions.*
